# Association of non-invasive liver-related scores with cardiovascular disease and a four-domain cardiovascular–renal–hepatic–metabolic phenotype in patients with type 2 diabetes mellitus and MASLD

**DOI:** 10.1007/s12020-026-04751-z

**Published:** 2026-08-15

**Authors:** Katerina Stefanaki, Dimitrios S. Karagiannakis, Maria Manolakopoulou, Stefania Magkou, Georgios Georgiopoulos, Michalis Liontos, Andromachi Vryonidou, Melpomeni Peppa, Evanthia Kassi, Theodora Psaltopoulou, Stavroula A. Paschou

**Affiliations:** 1 Endocrine Unit and Diabetes Center, Department of Clinical Therapeutics, School of Medicine, Alexandra Hospital, National and Kapodistrian University of Athens, Athens, Greece; 2 4th Department of Internal Medicine, School of Medicine, “Attikon” University Hospital, National and Kapodistrian University of Athens, Athens, Greece; 3 Department of Clinical Therapeutics, School of Medicine, Alexandra Hospital, National and Kapodistrian University of Athens, Athens, Greece; 4 School of Medicine, National and Kapodistrian University of Athens, Athens, Greece; 5 2nd Department of Cardiology, School of Medicine, “Attikon” University Hospital, National and Kapodistrian University of Athens, Athens, Greece; 6 Department of Endocrinology and Diabetes Center, Hellenic Red Cross Hospital, Athens, Greece; 7 Endocrine Unit, 2nd Dept of Internal Medicine-Propaedeutic, Research Institute and Diabetes Center, School of Medicine, National and Kapodistrian University of Athens, Athens, Greece; 8 1st Department of Propaedeutic Internal Medicine, School of Medicine, National and Kapodistrian University of Athens, Athens, Greece; 9 Imperial Center for Endocrinology, St Mary’s Hospital, Imperial College London, London, UK

**Keywords:** MASLD, Type 2 diabetes mellitus, Cardiovascular–kidney–liver–metabolic syndrome, FIB-4, Fibrotic NASH Index, The CORE model

## Abstract

**Objective:**

Metabolic dysfunction–associated steatotic liver disease (MASLD) is a systemic disorder associated with cardiovascular, renal, and metabolic comorbidities. We evaluated cardiovascular disease (CVD), four-domain cardiovascular–renal–hepatic–metabolic (CRHM) involvement, and their associations with non-invasive liver-related scores in patients with type 2 diabetes mellitus (T2DM) and MASLD.

**Methods:**

We retrospectively analyzed 216 patients with T2DM and MASLD. A study-specific four-domain CRHM phenotype was defined as the coexistence of T2DM, MASLD, chronic kidney disease (CKD), and atherosclerotic CVD. FIB-4, AST-to-ALT ratio (AAR), APRI, Hellenic Score II, Fibrotic NASH Index (FNI), and CORE model were evaluated.

**Results:**

CVD was present in 75/216 participants (34.7%). Among 206 participants with sufficient classification data, 35 (17.0%) had the four-domain CRHM phenotype. Patients with CVD had higher Hellenic Score II, creatinine, glycated hemoglobin, FNI, and CORE scores, and lower HDL. FNI showed modest discrimination for CVD (AUROC 0.65; 95%CI 0.56–0.74), while FNI combined with Hellenic Score II showed higher discrimination (AUROC 0.80; 95%CI 0.72–0.87). Participants with CRHM phenotype had higher APRI and FIB-4 and lower platelet counts. FIB-4 was independently associated with CRHM (OR 5.4; 95%CI 1.6–18.6) and showed moderate discriminative ability (AUROC 0.69; 95%CI 0.56–0.83). FIB-4 combined with CORE showed greater discriminative ability (AUROC 0.81; 95%CI 0.70–0.92).

**Conclusions:**

In patients with T2DM and MASLD, liver-related scores showed distinct associations with cardiovascular and multidomain disease burden. FNI was associated with CVD, whereas FIB-4 was associated with a study-specific four-domain CRHM phenotype. These findings are exploratory and require external validation.

**Supplementary Information:**

The online version contains supplementary material available at 10.1007/s12020-026-04751-z.

## Introduction

Metabolic dysfunction–associated steatotic liver disease (MASLD) has emerged as the most prevalent chronic liver disease worldwide, reflecting the growing burden of metabolic disorders in modern populations [1]. It is characterized by liver steatosis in the presence of at least one underlying cardiometabolic risk factor, such as obesity, type 2 diabetes mellitus (T2DM), dyslipidemia, or hypertension [2, 3].

Increasing evidence supports the recognition of MASLD as a hepatic manifestation of systemic metabolic dysfunction. It is closely linked to insulin resistance (IR) and is considered a key component of metabolic syndrome (MetS) [4]. Beyond its hepatic involvement, MASLD is associated with chronic, low-grade systemic inflammation. This persistent inflammatory milieu may contribute to endothelial dysfunction, promoting the development of atherosclerosis, and ultimately increasing the risk of cardiovascular diseases (CVD) [5] as well as chronic kidney disease (CKD) [6]. These interconnected pathophysiological processes highlight the systemic nature of MASLD and its role in the progression of multisystem disease.

In response to this growing understanding, the concept of the cardiovascular–kidney–liver–metabolic (CKLM) approach has been proposed to describe better the complex interplay among metabolic dysfunction, liver disease, and associated cardiovascular and renal complications [7]. This framework builds on and expands the earlier concept of the Cardiovascular–Kidney–Metabolic (CKM) syndrome, introduced in 2023 by the American Heart Association, which emphasized the interrelationships among metabolic risk factors, atherosclerotic disease, cardiovascular outcomes, and kidney dysfunction [8]. By incorporating hepatic involvement, the CKLM model provides a more comprehensive representation of the systemic consequences of metabolic disease [7]. More recently, the conceptual framework of the CKLM model has shifted toward an integrated approach, emphasizing staged risk assessment rather than binary disease classification and incorporating a more comprehensive evaluation of renal and hepatic involvement, including kidney function and albuminuria, as well as assessment of MASLD and liver fibrosis using non-invasive tests [9].

Among patients with MASLD, liver fibrosis constitutes a critical determinant of long-term prognosis. The progression from simple steatosis to fibrosis significantly increases the risk of cirrhosis, hepatic decompensation, and adverse liver-related outcomes [10]. In addition, liver fibrosis has been associated with an increased risk for cardiovascular events, renal impairment, and all-cause mortality [11, 12]. Although only approximately 5% of individuals with MASLD in primary care settings develop clinically significant fibrosis, this prevalence rises substantially—up to 20–25%—among patients with T2DM [13]. This subgroup is therefore at particularly high risk of developing complications within the spectrum.

In light of these associations, characterizing the relationship between metabolic dysfunction, liver-related abnormalities, and multisystem comorbidity remains clinically relevant. In this context, we aimed to evaluate the burden of established CVD and a study-specific four-domain phenotype comprising concomitant cardiovascular, renal, hepatic, and metabolic (CRHM) involvement among individuals with T2DM and MASLD. Furthermore, we aimed to investigate the associations between multiple non-invasive liver-related serum biomarkers and both CVD and the four-domain CRHM phenotype at the time of evaluation. An additional exploratory objective was to assess the discriminative ability of these non-invasive scores for identifying patients with multidomain disease.

## Materials and methods

### Patients

We retrospectively analyzed data from 238 consecutive patients aged 18 to 75 years who presented to our diabetes center between 01/09/2024 and 31/08/2025. We excluded subjects with a history of any chronic liver disease other than MASLD, excessive alcohol consumption (> 30 g per day for men and > 20 g per day for women) [2], inherited metabolic liver diseases, or use of medications that could cause hepatic disorders. Thus, from the initial cohort of patients, four were excluded due to alcohol drinking, and 18 due to the absence of liver steatosis on liver ultrasound.

## Methods

We reported epidemiological and somatometric characteristics and measured baseline blood parameters. A history of coronary artery disease, peripheral arteriopathy, or ischemic stroke defined CVD. Heart failure (HF) was excluded from the CVD definition in this study, despite its frequent inclusion in cardiovascular outcome studies, because HF represents a heterogeneous clinical syndrome with multiple etiologies and is not exclusively attributable to atherosclerotic CVD. Because CKLM has been proposed as a multidimensional, staged conceptual framework rather than as a binary diagnostic entity, we did not seek to establish a diagnostic definition of CKLM. For the purposes of the present exploratory analysis, we identified a study-specific four-domain CRHM phenotype characterized by the coexistence of four domains: T2DM, MASLD, CKD, and established atherosclerotic CVD. MASLD was diagnosed based on ultrasonographic evidence of liver steatosis after exclusion of alternative causes of liver disease. CKD was defined as an estimated glomerular filtration rate (eGFR) ≤ 60 mL/min/1.73 m², calculated using the 2021 CKD-EPI creatinine equation, that persisted for at least 3 months, according to current KDIGO recommendations [14]. Established atherosclerotic CVD was defined as a history of coronary artery disease, peripheral arterial disease, and/or ischemic stroke. None of the non-invasive liver-related scores evaluated in the present study was incorporated into the definition of this phenotype or used for patient classification. This study-specific phenotype was not intended to reproduce or replace contemporary CKLM staging frameworks but rather to characterize concomitant involvement across the four domains using the clinical variables available in this retrospective cohort.

Baseline Fibrosis-4 index (FIB-4) [15], aspartate aminotransferase (AST) to alanine aminotransferase (ALT) ratio (AAR) [16], AST/Platelets ratio (APRI) [17], Hellenic score II [18], Fibrotic NASH Index (FNI) [19], and the CORE (Cirrhosis Outcome Risk Estimator) model [20] were also evaluated. FIB-4 was calculated as: age × AST / [platelet count × √ALT] [15]. APRI was calculated as: [(AST / upper limit of normal AST) / platelet count] ×100 [17]. The Hellenic score II was calculated according to the original model by Panagiotakos et al. [18]. The FNI was calculated using AST, high-density lipoprotein (HDL), triglycerides (Tg), and glycosylated hemoglobin (HbA1c) values [19]. The CORE model was calculated using patient age, sex, and ALT, AST, and gamma-glutamyl transferase (GGT) values [20].

The protocol followed the Declaration of Helsinki (1983 revision) and was approved by the institution’s ethics committee. All participants provided informed consent for the use of anonymous data.

### Statistical Analysis

Data were analyzed using SPSS version 30 (SPSS Inc., Chicago, IL, USA). The Kolmogorov-Smirnov and Shapiro-Wilk tests were used to assess the normality of the variables’ distributions. Continuous variables with normal distributions were reported as mean ± standard deviation (SD); otherwise, as median ± interquartile range (IQR). Categorical variables were reported as counts and percentages (%). Quantitative variables with normal distributions were compared using the T-test; otherwise, the Mann-Whitney test was used. Qualitative variables were compared using the corrected chi-squared test or Fisher’s exact test, as appropriate. Univariate logistic regression analyses were performed to investigate the associations between variables and the presence of CVD or the four-domain CRHM phenotype. Variables with p-values < 0.05 were subsequently entered into a forward stepwise multivariate logistic regression, and odds ratios (ORs) with 95% confidence intervals (CIs) were reported. Given the limited number of participants exhibiting the four-domain CRHM phenotype (*n* = 35), the multivariable analyses were considered exploratory. The data-driven variable-selection procedure and the limited number of outcome events may increase the risk of model instability and overfitting; therefore, the adjusted estimates were interpreted with caution. Receiver operating characteristic (ROC) analysis was used to assess the discriminative ability of non-invasive serological tests for CVD and CRHM, and the Youden index was used to determine the exploratory cutoff for each test. Combined ROC analyses were based on predicted probabilities derived from binary logistic regression models. Because both cut-off derivation and discriminative performance assessment were conducted in the same cohort without external validation, the resulting AUROCs and cut-offs must be considered as exploratory. As an internal assessment of regression-coefficient stability, logistic regression models were additionally evaluated using 1,000 bootstrap resamples. Bootstrap resampling was not considered a substitute for external validation or formal assessment of model calibration. All tests were two-sided, and p-values < 0.05 were considered statistically significant.

## Results

A cohort of 216 consecutive patients with T2DM and MASLD was enrolled. The median age was 69.5 (14.8) years. The median duration of T2DM was 8.5 (10.4) years. The median values of FIB-4, APRI, FNI, and the CORE model were 1.03 (0.82), 0.19 (0.16), 0.18 (0.27), and 0.20 (0.14), respectively. The mean value of AAR was 0.98 ± 0.33. The remaining baseline characteristics are shown in Table 1.

**Table 1:** Patients baseline characteristics

Variable	Value
Age (years)	69.5 (14.8)
BMI (kg/m²)	31.3 ± 5.3
Diabetes duration (years)	8.5 (10.4)
GLP-1 therapy duration (years)	1.4 (1.7)
SGLT2 therapy duration (years)	1.4 (1.9)
Hellenic Score II	5.3 (4.8)
HbA1c (%)	6.9 (2.6)
TSH (mIU/L)	2.1 (1.0)
Cr (mg/dL)	0.9 (0.4)
AST (U/L)	19.0 (8)
ALT (U/L)	20.4 (13.4)
GGT (U/L)	22.3 (17.2)
^**^LDL (mg/dL)	84.5 (56.6)
HDL (mg/dL)	42.5 (17.6)
Tg (mg/dL)	155.0 (104)
Platelets (×10^3^/µL)	243.0 (130.0)
APRI	0.19 (0.16)
FIB-4	1.03 (0.82)
AAR	0.98 ± 0.33
FNI	0.18 (0.27)
CORE model (%)	0.20 (0.14)

^*^Values are expressed as mean ± standard deviation (SD), or median and interquartile range (IQR)

^**^Values under treatment with statins

BMI: Body mass index; GLP-1: Glucagon-like peptide-1; SGLT-2: Sodium glucose co-transporter 2; HbA1c: Glycosylated hemoglobulin; TSH: Thyroid stimulating hormone; Cr: Creatinine; AST: Aspartate aminotransferase; ALT: Alanine aminotransferase; GGT: Gamma glutamyl transferase; LDL: Low density lipoprotein; HDL: High density lipoprotein; Tg: Triglycerides; APRI: AST/Platelets ratio; FIB-4: Fibrosis 4 index; AAR: AST/ALT ratio; FNI: Fibrotic NASH index; CORE model: Cirrhosis outcome risk estimator model

Seventy-five (34.7%) patients had CVD (85.3% male; *n* = 64), and 141 (65.3%) did not (66.7% male; *n* = 94). Patients with CVD were significantly older [71.6 (10.6) vs. 66.7 (15.9) years; *p* = 0.01], with a significantly longer diabetes duration [10.3 (10.1) vs. 7.0 (12.2) years; *p* = 0.01], and had significantly higher Hellenic II score [5.9 (3.8) vs. 4.3 (5.5); *p* = 0.003], creatinine (Cr) levels [1.1 (0.4) vs. 0.8 (0.3); *p* < 0.001], HbA1c [7.5 (2.4) vs. 6.7 (2.5); *p* = 0.049], FNI [0.26 (0.28) vs. 0.15 (0.23); *p* = 0.002], and CORE model score [0.24 (0.14) vs. 0.19 (0.11); *p* = 0.011], while it had a significantly lower HDL levels [38.3 (16.8) vs. 45.0 (15.7) mg/dl; *p* = 0.001] than the non-CVD group. No significant differences were observed between the two groups in FIB-4, APRI, and AAR (Table 2).

**Table 2:** Comparison between patients without and with CVD

Variable	Non-CVD (*n* = 141)	CVD (*n* = 75)	*p*-value
Age (years)	66.7 (15.9)	71.6 (10.6)	0.01
BMI (kg/m²)	32.2 ± 5.0	29.8 ± 5.6	0.073
Diabetes duration (years)	7.0 (12.2)	10.3 (10.1)	0.01
GLP-1 therapy duration (years)	1.1 (1.6)	1.8 (1.4)	0.016
SGLT2 therapy duration (years)	1.3 (1.8)	1.6 (1.9)	0.195
Hellenic Score II	4.3 (5.5)	5.9 (3.8)	0.003
HbA1c (%)	6.7 (2.5)	7.5 (2.4)	0.049
TSH (mIU/L)	2.1 (1.7)	2.1 (0.8)	0.903
Cr (mg/dL)	0.8 (0.3)	1.1 (0.4)	< 0.001
AST (U/L)	18.3 (7.6)	20.3 (7.6)	0.087
ALT (U/L)	19.3 (11.7)	22.0 (13.1)	0.178
GGT (U/L)	22.0 (15.3)	23.0 (18.8)	0.738
^**^LDL (mg/dL)	102.0 (50.7)	63.5 (41.0)	< 0.001
HDL (mg/dL)	45.0 (15.7)	38.3 (16.8)	0.001
Tg (mg/dL)	161.0 (114.8)	130.5 (98.0)	0.433
Platelets (×10³/µL)	255.0 (135.5)	234.5 (128.0)	0.445
APRI	0.17 (0.16)	0.20 (0.14)	0.126
FIB-4	0.94 (0.58)	1.10 (0.90)	0.172
AAR	1.00 ± 0.30	1.00 ± 0.30	0.845
FNI	0.15 (0.23)	0.26 (0.28)	0.002
CORE model (%)	0.19 (0.11)	0.24 (0.14)	0.011

^*^Values are expressed as mean ± standard deviation (SD), or median and interquartile range (IQR)

^**^Values under treatment with statins

CVD: Cardiovascular disease; BMI: Body mass index; GLP-1: Glucagon-like peptide-1; SGLT-2: Sodium glucose co-transporter 2; HbA1c: Glycosylated hemoglobulin; TSH: Thyroid stimulating hormone; Cr: Creatinine; AST: Aspartate aminotransferase; ALT: Alanine aminotransferase; GGT: Gamma glutamyl transferase; LDL: Low density lipoprotein; HDL: High density lipoprotein; Tg: Triglycerides; APRI: AST/Platelets ratio; FIB-4: Fibrosis 4 index; AAR: AST/ALT ratio; FNI: Fibrotic NASH index; CORE model: Cirrhosis outcome risk estimator model

Compared with the non-CVD group, patients with CVD were more likely to have an FNI score ≥ 0.33, the proposed cutoff for ruling in significant fibrosis (≥ F2) with specificity > 90% [18] (*p* = 0.016) (Suppl. Figure 1). Similarly, they showed a higher prevalence of Hellenic score II ≥ 5, the threshold associated with a high risk of CVD [17] (*p* = 0.021) (Suppl. Figure 2). However, there was no significant difference between the two groups in the proportions of patients with an FIB-4 ≥ 1.3 (*p* = 0.256), which is the cutoff for ruling out significant fibrosis [3]. Likewise, the two groups did not differ significantly in the proportion of patients with a CORE model score ≥ 0.4%, which is the threshold used to rule out significant liver fibrosis ≥ F2 [19] (*p* = 0.421).

Parameters that significantly differed between patients with and without CVD were included in the univariate analysis. Age, diabetes duration, Hellenic score II, Cr levels, HDL, and the FNI index were significantly associated with CVD. In the multivariate analysis, which was adjusted for age, diabetes duration, Hellenic score II, Cr, and FNI, the presence of CVD was independently associated with age, Cr, and FNI (Table 3). HDL was excluded from the multivariate analysis to prevent multicollinearity, as it is a key component of FNI.

**Table 3:** Univariate and multivariate analysis of the variables associated with CVD Univariate analysis Multivariate analysis

	Univariate analysis			Multivariate analysis		
Variables	OR	95% CI	*p* value	OR	95% CI	*p* value
Age (years)	1.04	1.00-1.07	0.015	1.1	1.03–1.17	0.005
Diabetes duration (years)	1.04	1.00-1.1	0.031	0.97	0.92–1.02	0.224
HbA1c (%)	1.1	0.9–1.3	0.351			
Hellenic score II	1.13	1.04–1.24	0.005	1.02	0.9–1.17	0.839
Cr (mg/dl)	4.2	1.46-12.0	0.008	4.4	1.01–18.7	0.048
HDL (mg/dl)	0.97	0.95–0.99	0.033			
FNI	11.8	1.8–77.5	0.01	107.2	5.6–2035	0.002
CORE model (%)	1.3	0.8-2.0	0.331			

CVD: Cardiovascular disease; OR: Odds ratio; 95% CI: 95% Confidence interval; GLP-1: Glucagon-like peptide-1; HbA1c: Glycosylated hemoglobin; Cr: Creatinine; HDL: High density lipoprotein; FNI: Fibrotic NASH index; CORE model: Cirrhosis outcome risk estimator model

The discriminative ability of FIB-4, AAR, APRI, FNI, the CORE model, and Hellenic score II for the presence of CVD was evaluated using ROC curve analysis. FIB-4, AAR, and APRI did not demonstrate statistically significant discriminatory ability. FNI demonstrated modest discriminatory ability, yielding an AUROC of 0.65 (95% CI: 0.56–0.74; *p* = 0.001) for CVD. The exploratory cutoff was 0.23, with a sensitivity of 59% and a specificity of 72.2%. Likewise, the CORE model demonstrated an AUROC of 0.64 (95% CI: 0.54–0.74; *p* = 0.009) for CVD, with an exploratory cutoff of 0.27, yielding a sensitivity of 41.4% and a specificity of 82.1%. The Hellenic score II had an AUROC of 0.64 (95% CI: 0.55–0.73; *p* = 0.002) for CVD. The exploratory cutoff was 4, with a sensitivity of 86% and a specificity of 46.3%.

**Fig. 1:**
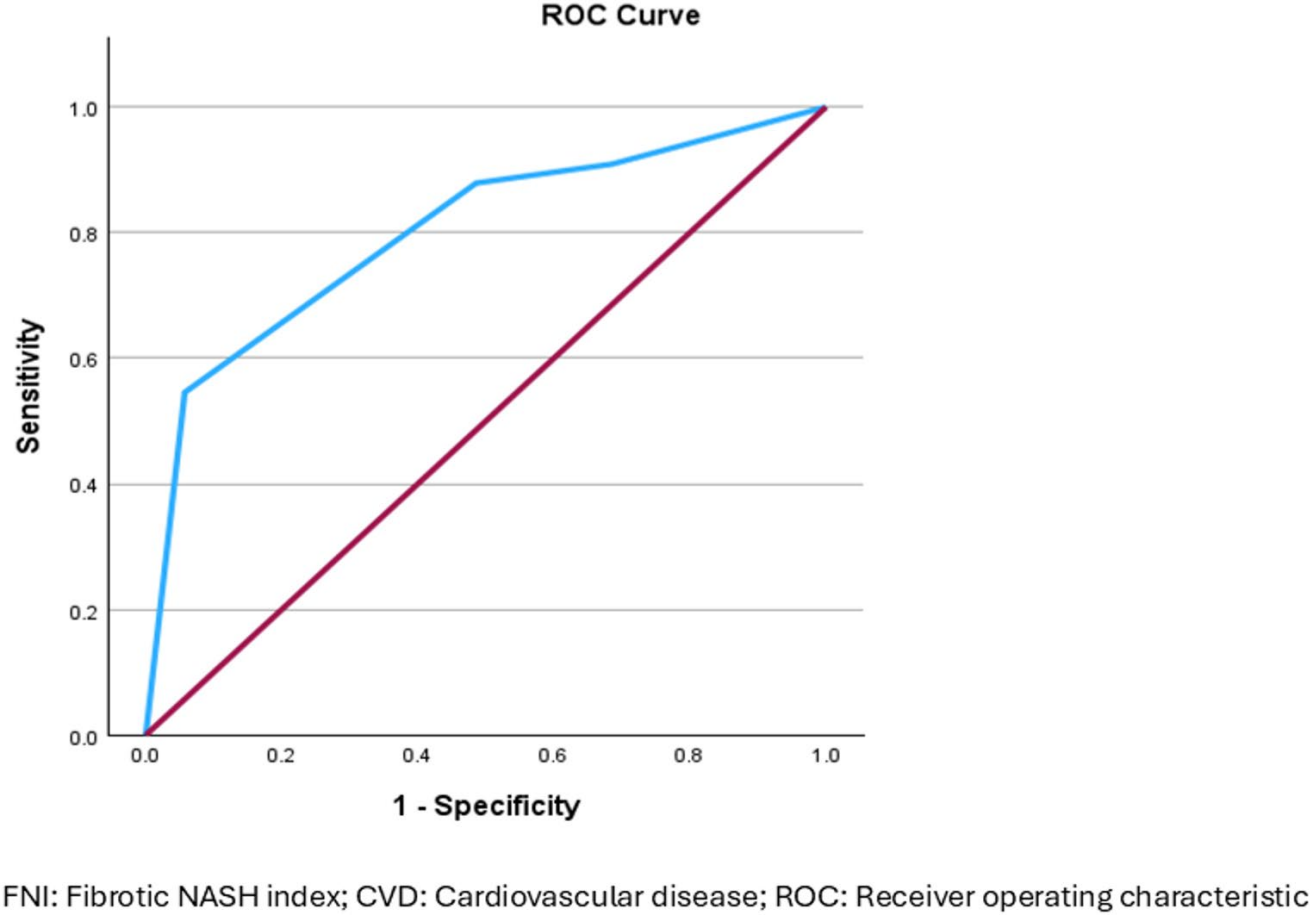
Discriminative ability of FNI ≥ 0.23 and Hellenic score II ≥ 4 for CVD

A combined model incorporating both FNI ≥ 0.23 and Hellenic score II ≥ 4, derived from logistic regression (FNI ≥ 0.23: OR 4.7; 95% CI 2.1–10.8; *p* < 0.001; Hellenic score II ≥ 4: OR 8.9; 95% CI 3.5–23.1; *p* < 0.001), yielded an improved AUROC of 0.8 (95% CI: 0.72–0.87; *p* < 0.001), indicating enhanced discriminative accuracy compared with either marker alone (Fig. 1). In an exploratory bootstrap analysis using 1,000 resamples, the regression coefficients for FNI ≥ 0.23 and Hellenic Score II ≥ 4 showed limited bias (0.073 and 0.123, respectively), with bias-corrected and accelerated 95% CIs remaining above zero, supporting the internal stability of these associations.

Of the 206 participants with complete classification data, 35 (17.0%) fulfilled the criteria for the four-domain CRHM phenotype, whereas 171 (83%) did not. Ten participants could not be classified because renal function data were unavailable due to the study’s retrospective design, rather than to predefined clinical characteristics or selective exclusion. Compared with patients without the four-domain CRHM, those with CRHM were older [71.4 (10.8) vs. 66.7 (15.9) years; *p* = 0.053], had significantly higher Cr [1.2 (0.2) vs. 0.8 (0.3); *p* < 0.001], and HbA1c [7.5 (2.1) vs. 6.7 (2.5) %; *p* = 0.025], significantly lower platelet counts [226.0 (67.5) vs. 255.0 (135.5) ×10³/µL; *p* = 0.01], and significantly higher APRI [0.24 (0.17) vs. 0.17 (0.16); *p* = 0.02] and FIB-4 [1.66 (1.07) vs. 0.94 (0.58); *p* = 0.002] scores, while it showed a trend towards a higher CORE model [0.27 (0.10) vs. 0.19 (0.11) %; *p* = 0.062] (Table 4).

**Table 4:** Comparison between patients without and with CRHM

Variable	Non-CRHM (*n* = 171)	CRHM (*n* = 35)	*p*-value
Age (years)	66.7 (15.9)	71.4 (10.8)	0.053
BMI (kg/m²)	31.5 ± 5.2	31.7 ± 5.6	0.894
Diabetes duration (years)	7.0 (12.2)	9.0 (9.9)	0.346
GLP-1 therapy duration (years)	1.1 (1.6)	1.7 (2.9)	0.25
SGLT2 therapy duration (years)	1.3 (1.8)	1.6 (2.2)	0.682
Hellenic Score II	4.3 (5.5)	5.3 (3.6)	0.284
HbA1c (%)	6.7 (2.5)	7.5 (2.1)	0.025
TSH (mIU/L)	2.0 (1.7)	2.2 (1.5)	0.456
Cr (mg/dL)	0.8 (0.3)	1.2 (0.2)	< 0.001
AST (U/L)	18.3 (7.6)	22.5 (11.7)	0.094
ALT (U/L)	19.3 (11.7)	21.0 (18.7)	0.406
GGT (U/L)	22.0 (15.3)	19.5 (21.9)	0.506
^**^LDL (mg/dL)	102.0 (50.7)	63.0 (41.8)	< 0.001
HDL (mg/dL)	45.0 (15.7)	40.0 (19.1)	0.573
Tg (mg/dL)	161.0 (114.8)	149.5 (111.0)	0.807
Platelets (×10³/µL)	255.0 (135.5)	226.0 (67.5)	0.01
APRI	0.17 (0.16)	0.24 (0.17)	0.02
FIB-4	0.94 (0.58)	1.66 (1.07)	0.002
AAR	0.95 ± 0.30	1.00 ± 0.30	0.272
FNI	0.15 (0.23)	0.25 (0.30)	0.113
CORE model (%)	0.19 (0.11)	0.27 (0.10)	0.062

^*^Values are expressed as mean ± standard deviation (SD), or median and interquartile range (IQR)

^**^Values under treatment with statins

CRHM: Cardiovascular–renal–hepatic–metabolic domain; BMI: Body mass index; GLP-1: Glucagon-like peptide-1; SGLT-2: Sodium glucose co-transporter 2; HbA1c: Glycosylated hemoglobulin; TSH: Thyroid stimulating hormone; Cr: Creatinine; AST: Aspartate aminotransferase; ALT: Alanine aminotransferase; GGT: Gamma glutamyl transferase; LDL: Low density lipoprotein; HDL: High density lipoprotein; Tg: Triglycerides; APRI: AST/Platelets ratio; FIB-4: Fibrosis 4 index; AAR: AST/ALT ratio; FNI: Fibrotic NASH index; CORE model: Cirrhosis outcome risk estimator model

Compared with the non-CRHM group, the CRHM group was more likely to have FIB-4 ≥ 1.3 (*p* < 0.001) (Suppl. Figure 3), but there was no significant difference in the proportions of patients with an FNI ≥ 0.33, a Hellenic score II ≥ 5, or a CORE Model ≥ 0.4 between the two groups.

Parameters that differed significantly between patients with and without CRHM were included in the univariate analysis. Cr levels, APRI, and FIB-4 were all significantly associated with four-domain CRHM. Cr was excluded from the multivariate analysis because it is essential for diagnosing CKD, which is a component of CRHM. Although APRI was associated with CRHM in univariate analysis, this association did not persist after adjustment. Thus, in the multivariate analysis, only FIB-4 remained independently associated with CRHM; each 1-unit increase in FIB-4 was associated with a 5.4-fold increase in the odds of CRHM. (Table 5).

**Table 5:** Univariate and multivariate analysis of the variables associated with CRHM

	Univariate analysis			Multivariate analysis		
Variables	OR	95% CI	*p* value	OR	95% CI	*p* value
HbA1c (%)	1.1	0.9–1.4	0.36			
Cr	25.8	6.3-106.5	< 0.001			
Platelets (×10^3^/µL)	1.0	1.0–1.0	0.087			
APRI	50.7	2-1302.6	0.018	0.2	0.001–29.5	0.535
FIB-4	4.1	1.8–9.1	< 0.001	5.4	1.6–18.6	0.007

CRHM: Cardiovascular–renal–hepatic–metabolic domain; OR: Odds ratio; 95% CI: 95% Confidence interval; HbA1c: Glycosylated hemoglobin; Cr: Creatinine; APRI: Aspartate aminotransferase (AST)/Platelets ratio; FIB-4: Fibrosis-4 index

Again, the discriminative ability of FIB-4, AAR, APRI, FNI, the CORE model, and Hellenic score II for CRHM was evaluated using ROC curve analysis. The AUROCs for AAR, Hellenic score II, and FNI did not reach statistical significance. By contrast, FIB-4 demonstrated a moderate AUROC of 0.69 (95% CI: 0.56–0.83; *p* = 0.004) for CRHM. The exploratory cutoff was 1.54, with a sensitivity of 65.4% and a specificity of 83%. APRI offered an AUROC of 0.64 (95% CI: 0.52–0.76; *p* = 0.019) for CRHM. The exploratory cutoff was 0.25, with a sensitivity of 42.3% and a specificity of 79.4%. The CORE model yielded an AUROC of 0.64 (95% CI: 0.51–0.76; *p* = 0.036), with a sensitivity of 65% and a specificity of 63.1% at the exploratory cutoff of 0.2.

**Fig. 2:**
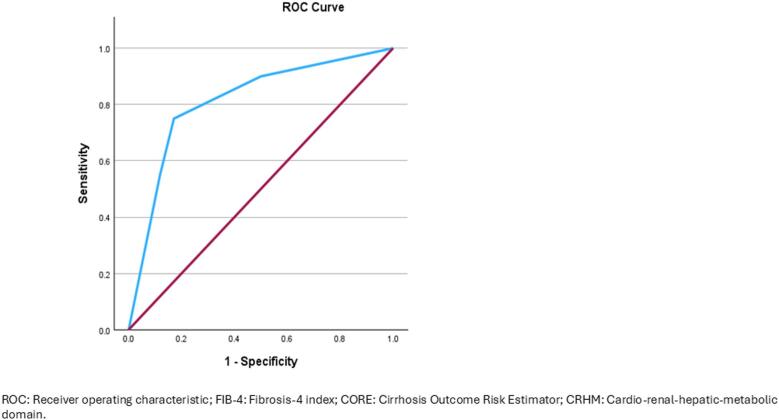
ROC Curve for FIB-4 ≥ 1.54 and CORE model ≥ 0.2 for the four-domain CRHM phenotype

A combined model incorporating both FIB-4 ≥ 1.54 and CORE model ≥ 0.2, derived from logistic regression (FIB-4 ≥ 1.54: OR 12.9; 95% CI 3.8–43; CORE model ≥ 0.2: OR 1.6; 95% CI 0.5–5.5), achieved the highest AUROC of 0.81 (95% CI 0.70–0.92; *p* < 0.001) (Fig. 2). In the corresponding bootstrap analysis with 1,000 resamples, FIB-4 ≥ 1.54 showed limited coefficient bias (0.208), and its bias-corrected and accelerated 95% CI remained above zero. In contrast, the exploratory bootstrap 95% CI for CORE ≥ 0.20 crossed zero, indicating greater uncertainty about CORE’s independent contribution to the combined model.

## Discussion

MASLD has emerged as the most prevalent liver disease globally [1]. It encompasses a broad pathological spectrum, ranging from simple steatosis to steatohepatitis, liver fibrosis, and cirrhosis [3]. Increasing evidence supports the conceptualization of MASLD as a systemic disease rather than a condition exclusively confined to the liver, which is associated with an elevated risk of CVD and CKD [5, 6]. IR and MetS, the principal underlying pathophysiological features of MASLD, contribute to low-grade systemic inflammation [4]. This inflammatory milieu extends beyond hepatic inflammation and may contribute to endothelial dysfunction and atherosclerosis, thereby exacerbating the risk of both CVD and CKD [4]. This interplay among MetS, MASLD, CVD, and CKD is currently acknowledged as CKLM [9]. Except for systemic inflammation, liver fibrosis appears to play a pivotal role in this continuum, serving as an independent risk factor for cirrhosis, CVD, and CKD [10–12]. Consequently, identifying biomarkers that reflect both hepatic injury and the broader multisystem disease burden has become a priority in clinical research [21, 22].

In this study, we examined the association of liver-related non-invasive scores with CVD and a study-specific four-domain CRHM phenotype in individuals with T2DM and MASLD. In addition to routinely used scores such as FIB-4, APRI, and AAR, we assessed the discriminative utility of recently developed composite scores. We included the FNI, which incorporates both liver and metabolic parameters and thus likely reflects the underlying predisposing factors for MASLD better [19]. Furthermore, we applied the CORE model, which has been shown to correlate with the development of liver-related events [20].

In a cohort of 216 participants, approximately one-third had CVD. Individuals with CVD had a longer duration of T2DM and worse glycemic control. They also had higher Hellenic score II values and lower HDL concentrations. Elevated Cr levels were noted, suggesting increased susceptibility to CKD and, consequently, the four-domain CRHM. Patients with CVD exhibited significantly lower LDL cholesterol levels and a longer duration of treatment with GLP-1 analogs or SGLT2 inhibitors compared with those without CVD. However, these findings should be interpreted with caution because both variables are highly susceptible to confounding by indication. Current international guidelines strongly recommend intensive lipid-lowering therapy, predominantly with statins, for patients with established CVD or those considered at high cardiovascular risk [23]. Consequently, lower LDL levels in the CVD group are likely to reflect more aggressive therapeutic intervention rather than an intrinsically lower cardiovascular risk profile. In this context, LDL cholesterol may act as a marker of treatment intensity rather than a true biological determinant of cardiovascular outcomes. A similar consideration applies to treatment with GLP-1 analogs or SGLT2 inhibitors. Evidence from large cardiovascular outcome trials has demonstrated the cardioprotective and renal protective benefits of these agents in patients with T2DM, particularly among those with established atherosclerotic CVD or multiple cardiovascular risk factors [24, 25]. As a result, these medicines are preferentially prescribed to patients with CVD or at higher cardiovascular risk. Therefore, the longer treatment duration of GLP-1 analogs or SGLT-2 inhibitors observed among patients with CVD most likely reflects the implementation of contemporary guideline-directed therapy rather than a causal relationship between treatment exposure and the presence of CVD. Because both LDL levels and the duration of GLP-1 analog or SGLT2 inhibitor therapy are strongly influenced by clinical decision-making and treatment allocation, their inclusion in regression models could introduce substantial bias and obscure the relationship between liver-related biomarkers and cardiovascular outcomes. Given their strong dependence on treatment allocation and clinical indication, these variables were not included in the primary multivariable models; nevertheless, residual treatment-related confounding cannot be excluded. In particular, detailed information regarding statin type, dose, intensity, and duration was not consistently available. Moreover, GLP-1 receptor agonists and SGLT2 inhibitors may influence cardiovascular and renal outcomes themselves, while being preferentially prescribed to patients with established CVD, CKD, or higher cardiorenal risk. Thus, the observed treatment differences may reflect both treatment allocation based on baseline risk and potential treatment effects and should not be interpreted as causal associations with the clinical phenotypes examined.

Notably, CVD was significantly associated with the newer composite scores, namely the FNI and the CORE model, but not with traditional markers such as FIB-4, APRI, and AAR. In addition, patients with CVD were more likely to have an FNI ≥ 0.33 than those without CVD, whereas no significant difference was observed in the proportion of patients with FIB-4 ≥ 1.3. In the multivariate analysis, age, Cr, and FNI were the only variables independently associated with CVD. However, the association between FNI and CVD was characterized by a wide confidence interval. This likely reflects the narrow distribution of FNI values within the study population and the relatively small number of patients with high FNI values. Therefore, although FNI remained independently associated with CVD, the magnitude of the observed effect should be interpreted with caution.

The differing compositions of FNI and FIB-4 may partly account for their distinct associations with CVD. FNI incorporates several metabolic variables, including BMI, HDL cholesterol, and HbA1c, which are themselves closely associated with cardiovascular risk. Therefore, the observed association between FNI and CVD may reflect, at least in part, the broader cardiometabolic information incorporated into the score rather than a specific metabolic-inflammatory mechanism. The absence of associations between CVD and FIB-4, APRI, or AAR is noteworthy but does not establish that metabolic, rather than fibrosis-related, pathways underlie the observed findings. Indeed, the present study did not directly assess systemic inflammation, endothelial dysfunction, or liver fibrosis. Therefore, any biological explanation for the differential associations of these scores remains hypothesis-generating.

Interestingly, combining FNI with Hellenic Score II yielded higher discrimination for CVD than either score alone. However, both scores incorporate HDL cholesterol, resulting in partial structural overlap. Although FNI and Hellenic Score II were not significantly correlated in our cohort (rho = 0.083, *p* = 0.339; data not shown), this does not establish their independence, as the lack of a significant correlation may reflect contributions from other, non-overlapping components apart from HDL. Therefore, the higher discrimination of the combined model should be interpreted cautiously, and its incremental value requires confirmation in an independent cohort. Furthermore, because the cut-offs were derived and their discriminatory performance evaluated in the same cohort, the observed AUROCs are considered exploratory.

Regarding patients with the four-domain CRHM phenotype, the FIB-4 and APRI scores were significantly elevated, and platelet counts were notably lower than in individuals without CRHM. Patients with CRHM also tended to have higher CORE scores than those without CRHM, but the difference did not reach statistical significance (*p* = 0.062). Additionally, subjects with CRHM had FIB-4 scores ≥ 1.3 more often than those without CRHM. The multivariate analysis confirmed a significant association between FIB-4 and CRHM, with a 5.4-fold increase in the odds of CRHM per 1-unit increase in FIB-4. In contrast, APRI was no longer significantly associated with CRHM after adjustment, suggesting that FIB-4 may provide more robust information regarding the fibrosis-related component of CRHM in this cohort. Interestingly, the addition of the CORE model to FIB-4 resulted in numerically improved discriminative ability for CRHM compared with FIB-4 alone (AUROC 0.81 vs. 0.69, respectively). Bootstrap resampling provided some evidence of internal stability in the observed associations, particularly for FNI, Hellenic Score II, and FIB-4, whereas CORE’s contribution to the combined model was less stable. However, bootstrap assessment of coefficient stability does not establish model calibration or external validity. Accordingly, the observed discriminative performance and derived cut-offs should be considered exploratory and should be validated in independent cohorts.

The association between FIB-4 and the four-domain CRHM phenotype should be interpreted cautiously. Although FIB-4 is widely used for risk stratification of advanced liver fibrosis, it incorporates age, AST, ALT, and platelet count, variables that are not specific to hepatic fibrosis and may themselves be associated with cardiovascular, renal, or systemic disease. Thus, higher FIB-4 values among participants with the four-domain CRHM phenotype may reflect a combination of hepatic and extrahepatic factors rather than liver fibrosis alone. This consideration is particularly relevant because FIB-4, as well as the other non-invasive scores evaluated in the present study, is a surrogate marker rather than a direct measure of liver fibrosis. Since liver elastography, other imaging-based fibrosis assessments, and liver histology were not available in our cohort, we cannot determine whether the observed associations are driven by true differences in fibrosis severity or by contributions from individual score components. Consequently, the biological mechanisms underlying the observed association cannot be established from the present data, and any interpretations should be considered hypothesis-generating rather than conclusive and should be confirmed in future mechanistic and longitudinal studies.

The absence of an association between FIB-4 and CVD in the present study appears inconsistent with our previous findings [21]. However, important methodological differences may explain this discrepancy. In the earlier study, the CVD group also included patients with HF. In contrast, HF was excluded from the current analysis because it is a heterogeneous clinical syndrome not exclusively attributable to atherosclerotic disease. Furthermore, kidney function was not assessed in the previous cohort; therefore, some patients classified as having CVD may also have had concomitant CKD and would potentially have fulfilled the four-domain CRHM phenotype. These differences in definitions and patient characterization may partly explain the differing associations observed between FIB-4 and CVD across the two studies [21].

Our findings highlight the substantial burden of multisystem comorbidity in patients with T2DM and MASLD. In this selected cohort, approximately 17% of evaluable participants exhibited concomitant metabolic, hepatic, renal, and cardiovascular involvement, as defined by our study-specific four-domain CRHM phenotype. Because T2DM and MASLD were inclusion criteria, this phenotype primarily identified patients in whom CKD and established atherosclerotic CVD additionally coexisted. Our study-specific four-domain phenotype should be distinguished from the contemporary CKM and emerging CKLM frameworks, which provide broader, more granular approaches to multidomain risk assessment [9]. In particular, these frameworks incorporate earlier stages of cardiometabolic risk and more comprehensive characterization of renal and hepatic involvement, including albuminuria and non-invasive assessment of liver fibrosis [9]. Our retrospective dataset did not permit complete implementation of such a framework; therefore, the four-domain phenotype used in the present study should be regarded as a simplified exploratory construct rather than an alternative staging system.

An important potential clinical implication of our findings is that liver-related non-invasive scores routinely obtained in patients with T2DM and MASLD may provide information that extends beyond their conventional hepatic applications. These scores are simple and inexpensive to calculate and are based on clinical and laboratory parameters that are readily available in routine practice, without requiring additional specialized investigations. Their potential value in this context would not be to replace direct identification of established CVD or CKD, but rather to provide an additional signal of the broader multisystem disease burden associated with MASLD. In particular, the association of FNI with established CVD and of FIB-4 with concomitant cardiovascular and renal involvement suggests that different scores may capture partly different dimensions of this burden. In clinical settings where patients with T2DM and MASLD are often managed across different specialties, an elevated liver-related score could prompt a more comprehensive assessment of cardiovascular and renal comorbidities rather than being interpreted solely within a hepatic context. Moreover, the higher apparent discrimination observed with combined models suggests that integrating complementary, readily available non-invasive markers may ultimately yield a more informative multidomain assessment than individual scores considered in isolation. However, the present study does not demonstrate that such an approach improves the detection of previously unrecognized disease, clinical decision-making, or patient outcomes, and its potential clinical utility requires prospective validation. Furthermore, these markers were evaluated only in relation to the presence of CVD and CRHM at the time of recruitment, rather than as predictors of future outcomes. Therefore, no conclusions can be drawn about their prognostic value for cardiovascular, renal, or hepatic events. Future longitudinal studies are needed to determine whether these markers can reliably predict disease progression and adverse outcomes across the CRHM spectrum.

Several limitations should be acknowledged. First, liver fibrosis was not assessed with imaging-based methods, such as transient elastography or magnetic resonance elastography, and histological confirmation was unavailable. Consequently, fibrosis risk was evaluated exclusively using serum-based non-invasive scores, and the presence or severity of hepatic fibrosis could not be directly confirmed, as the non-invasive tests used are surrogate markers rather than direct measures of fibrosis. In addition, albuminuria was not systematically available, and renal involvement was therefore defined according to persistent reduction in eGFR. Consequently, our study-specific four-domain phenotype does not capture the full spectrum of renal involvement incorporated into contemporary multidomain CKLM frameworks. Second, the retrospective cross-sectional design precludes causal inference and is susceptible to residual confounding. Furthermore, because liver-related biomarkers and clinical outcomes were assessed at the same point, the possibility of reverse causation cannot be excluded. Therefore, the observed associations should not be interpreted as evidence of a temporal or causal relationship between liver-related scores and CVD or the four-domain CRHM. Third, detailed information regarding statin type, dose, intensity, and treatment duration was not consistently available, preventing adequate adjustment for lipid-lowering therapy. The lower LDL cholesterol levels observed among participants with CVD and the four-domain CRHM phenotype may therefore reflect differences in treatment intensity rather than differences in underlying cardiovascular risk. Similarly, GLP-1 receptor agonists and SGLT2 inhibitors are preferentially prescribed to patients with established or increased cardiovascular and renal risk and may themselves modify cardiorenal outcomes. Consequently, confounding by indication and residual treatment-related confounding cannot be excluded. Fourth, the relatively small number of participants exhibiting the four-domain CRHM phenotype (*n* = 35) represents an important limitation. This, together with the data-driven selection of candidate variables for multivariable modeling, may have increased the risk of model instability and overfitting. The wide confidence intervals observed for some estimates further indicate substantial imprecision. Therefore, the multivariable findings should be considered exploratory and hypothesis-generating and should be confirmed in larger independent cohorts. Fifth, the discriminatory performance of the individual and combined scores was evaluated in the same cohort from which the cut-offs were derived, using the Youden index; therefore, the reported AUROCs and cut-offs may be considered exploratory. Although bootstrap resampling was performed as an internal assessment of regression-coefficient stability, it does not replace external validation or formal assessment of model calibration. Consequently, the reported cut-offs and discriminatory estimates should not be applied clinically without validation in independent cohorts. Sixth, there is some overlap between the FNI and the Hellenic Score II because both scores incorporate HDL cholesterol. Although these models also contain distinct variables, part of the improved discriminative ability observed for the combined model may reflect information shared by this common component. Finally, our study population comprised patients with established T2DM and MASLD who were evaluated at a tertiary referral center. Consequently, the proportion of participants exhibiting the four-domain CRHM phenotype should not be interpreted as the prevalence of an established syndrome or generalized to broader populations.

In conclusion, this study supports the view that MASLD in patients with T2DM may occur within a broader context of cardiovascular, renal, hepatic, and metabolic comorbidities. Our findings suggest that liver-related non-invasive scores may be differentially associated with aspects of this multisystem disease burden. In particular, FNI was associated with established CVD, whereas FIB-4 was independently associated with the study-specific four-domain CRHM phenotype. Moreover, the higher apparent discrimination observed with combined models suggests that integrating multiple non-invasive markers may improve the assessment of cardiovascular and multidomain disease burden compared with individual scores alone. However, these findings should be considered exploratory and hypothesis-generating, given the cross-sectional design, the limited number of participants with four-domain involvement, the lack of direct evaluation of liver fibrosis, and the absence of external validation. Independent validation in larger prospective cohorts is required before any clinical implications can be drawn.

## Supplementary Information

Below is the link to the electronic supplementary material.

Supplementary Material 1

Supplementary Material 2

Supplementary Material 3
